# Retracted: Observation of the Auxiliary Treatment Effect of Low-Frequency Nerve Therapy Instrument after Hysteroscopy for Moderate and Severe Intrauterine Adhesions Based on Intelligent Medical Treatment

**DOI:** 10.1155/2022/9790264

**Published:** 2022-11-23

**Authors:** Journal of Healthcare Engineering

*Journal of Healthcare Engineering* has retracted the article titled “Observation of the Auxiliary Treatment Effect of Low-Frequency Nerve Therapy Instrument after Hysteroscopy for Moderate and Severe Intrauterine Adhesions Based on Intelligent Medical Treatment” [1] due to concerns that the peer review process has been compromised.

Following an investigation conducted by the Hindawi Research Integrity team [2], significant concerns were identified with the peer reviewers assigned to this article; the investigation has concluded that the peer review process was compromised. We therefore can no longer trust the peer review process, and the article is being retracted with the agreement of the Chief Editor.
